# Training to reduce emergency responders’ perceived overdose risk from contact with fentanyl: early evidence of success

**DOI:** 10.1186/s12954-020-00402-2

**Published:** 2020-08-24

**Authors:** Rachel P. Winograd, Sarah Phillips, Claire A. Wood, Lauren Green, Brandon Costerison, Jeremiah Goulka, Leo Beletsky

**Affiliations:** 1 Missouri Institute of Mental Health, University of Missouri, St. Louis, Missouri USA; 2 National Council on Alcoholism and Drug Abuse, St. Louis, Missouri USA; 3 Northeastern University, Boston, Massachusetts USA

## Introduction

Overdose deaths involving opioids in the USA have skyrocketed 41.5% since 2010 [1], with a provisional count of over 47,105 deaths from August 2018 to August 2019 [2]. Synthetic opioids, particularly fentanyl, have driven deaths in recent years due to their high potency and low overdose threshold. Indeed, death statistics in Missouri suggest over 75% of fatal opioid overdoses involved fentanyl in 2018 [3].

In Missouri, emergency medical services (EMS) personnel and law enforcement officers (LEOs) are called to respond to the increasing numbers of opioid overdoses. Emergency responders report frequent occupational safety concerns related to illicit drug use [4]. One common fear is needle stick injury and the subsequent potential risk of contracting infectious diseases [5]. Additionally, emergency responders express concern for their own safety during overdose reversals, including purported instances of aggression and combativeness on the part of the survivor [6, 7].

With the relatively recent surge in fentanyl-related overdoses, a new occupational safety concern has emerged among emergency responders: the fear of overdosing from touching fentanyl [8]. In 2017 alone, over 150 media reports describing first responder exposures to opioids surfaced [9]. Reports of overdose due to fentanyl contact among first responders [10–13] have been repeatedly refuted by medical experts [14–16]. Yet, mixed messages from the US government agencies [17] and their prominence in media outlets have catalyzed the spread of misinformation about the risks of accidental fentanyl contact. The high level of concern about this theoretical threat has been especially stark in the context of the COVID-19 pandemic, particularly in the USA, when police have reportedly expressed comparatively little anxiety about contracting the potentially deadly virus [18].

There has been an increase in products marketed to address the fear of fentanyl, including fentanyl exposure prevention kits [19, 20], gloves marketed to protect against fentanyl [21], other fentanyl-resistant gear and screening devices [22], and fentanyl clean-ups [23]. Additionally, legislators in the USA have proposed the Providing Officers with Electronic Resources (POWER) Act that would fund state and local enforcement agencies to purchase fentanyl screening devices to protect officers from incidental exposure [24]. However, because these screening procedures require the use of class B hazmat suits [25] and other equipment prior to responding to the overdose, these precautions could potentially delay the time-sensitive, lifesaving administration of naloxone and rescue breathing.

Concerns about fentanyl exposure continue to spread despite a clear consensus from medical experts that overdose from incidental skin contact is a medical impossibility [14, 15]. Indeed, this claim has been officially debunked by the American College of Medical Toxicology and the American Academy of Clinical Toxicology [16] and the National Occupational Safety and Health with the CDC [26]. A drug policy advocate has also disproven this myth by holding fentanyl powder in his hand without consequence and widely circulating the internet footage [15]. Researchers who study reported overdoses from fentanyl exposure among emergency responders have explained that cases documented thus far can best be attributed to the “nocebo effect”—a phenomenon in which individuals believe they have encountered a toxic substance and therefore experience the expected symptoms of exposure [27]. This is consistent with our broader understanding of occupational wellness and mental health—or lack thereof—among first responders [28]. When individuals are already operating under acute stress and with few mental health reserves, fear of overdose from touching fentanyl could serve as an additional stressor.

To our knowledge, only one study has examined emergency responders’ perceptions of risk associated with brief exposure to fentanyl. In their study of 247 first responders in New York, Persaud and Jennings found 80% of responders believed “briefly touching fentanyl could be deadly.” Based on these findings, the authors concluded trainings should incorporate accurate information about fentanyl risk and overdose response. To date, no studies have explored the extent to which these beliefs are modifiable through training and education.

As part of a broader effort to improve community access to naloxone, the authors of the current study developed comprehensive training for emergency responders on recognizing and responding to an overdose, basic tenets of addiction, and the roles of treatment and harm reduction strategies. Because unfounded fears about incidental contact with fentanyl could result in delays in responding to overdoses, we incorporated medically accurate information regarding fentanyl exposure into this existing overdose education and response training program. In addition to increasing knowledge and improving attitudes towards overdose recognition and response scenarios [29, 30], we aimed to decrease participants’ endorsement of the pre- and post-training survey statement “I can overdose from touching fentanyl.”

## Methods

### Participants

Between January and August 2019, project staff conducted eight LEO trainings and three EMS trainings for a total of 11 trainings reaching 200 participants. All trainings were conducted in the Eastern Region of Missouri, which consistently demonstrates the highest drug overdose rates in the state [3]. Though all trainings were tailored for professional emergency responders, other professionals were allowed to attend, though their survey data was omitted (*n* = 17). For this study, 113 participants identified as an LEO or Security Officer “LEO group” and 27 participants identified as an EMS, EMT, or Fire Department Worker “EMS group”, for a total of 140 participants.

### Demographics

Demographic data were collected on the age, race, ethnicity, and gender of each participant (see Table 1).

**Table 1:** Demographics

	Overall (*N* = 140)	LEO group (*n* = 113)	EMS group (*n* = 27)
Mean age (SD)	40.96 (11.27)	40.6 (11.0)	42.3 (12.5)
Men	110 (79.7%)	98 (86.7%)	12 (44.4%)
Hispanic/Latino(a)	5 (3.6%)	4 (3.5%)	1 (3.7%)
Race			
White	115 (82.1%)	89 (78.8%)	26 (96.3%)
Black	15 (10.7%)	15 (13.3%)	0
Multiracial or other^1^	4 (2.9%)	3 (2.7%)	1 (3.7%)
Refused	6 (4.3%)	6 (5.3%)	0

^1^Other races includes Asian, American Indian/Alaskan Native, or Native Hawaiian/Pacific Islander

### Procedure

LEO and EMS training content included information about the nature of addiction as a chronic brain disease, harm reduction principles, concerns about “enabling” drug use (i.e., naloxone-related risk compensation [29]), the role of addiction treatment medications, and Missouri’s naloxone and Good Samaritan laws, in addition to accurate information regarding the medical impossibility of overdosing from incidental skin exposure to fentanyl. The training portion addressing fentanyl misconceptions took approximately 10 min to present and discuss. In LEO trainings, the fentanyl information was taught by an emergency medicine physician. For EMS trainings, it was covered by the Project Manager.

Participants were consented prior to the start of each training. They were then given a paper survey including items tapping their knowledge and attitudes about a range of topics related to opioid overdose recognition and response, including but not limited to their belief that it is possible to overdose from touching fentanyl (for the purposes of this study, only this item related to fentanyl contact was analyzed and reported). Participants completed the same survey immediately following the training. The surveys were later manually entered into REDCap by a research assistant. The University of Missouri–St. Louis IRB approved this research study.

### Measure of belief in fentanyl exposure risk

Before and after the training session, participants indicated their agreement with the following original item by checking either “True” or “False”: “I can overdose from touching fentanyl*.*”

### Statistical analyses

We used SPSS to conduct a Fisher’s exact test to assess differences in pre- and post-training item responses across the LEO and EMS groups. We then applied McNemar’s test, appropriate for paired, categorical data, to assess changes in the pre- and post-training proportions of responses in the overall sample and by professional groups (LEO vs. EMS) to determine the effectiveness of brief training on reducing the false belief that one can overdose from touching fentanyl.

## Results

### Baseline differences by profession

On the pre-training survey, only 20.9% of all participants correctly answered “False” to the statement “I can overdose from touching fentanyl.” There were differences in responses across the professional groups, with 16.8% of LEOs and 37.0% of EMS participants answering “False” (Fisher’s exact test; *p* = .033).

### Effect of brief training

**Fig. 1:**
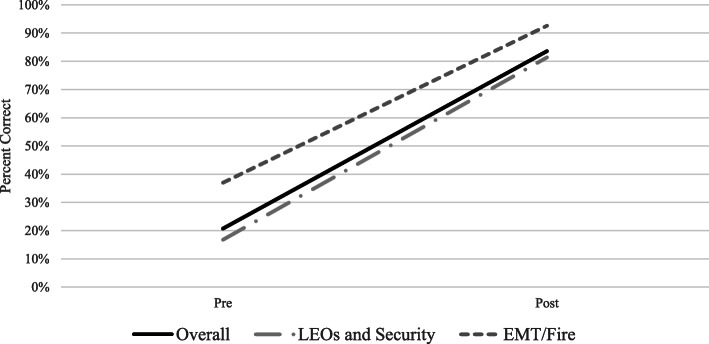
Percent correctly responding “False” to the item “I can overdose from touching fentanyl” at pre-training and post-training

Post-training, 83.6% of all participants correctly responded “False” to the statement (a difference of 62.7% from pre-training; McNemar’s test; *p* < .001). Within group pre-post comparisons also reached significance for both groups, with 81.4% of the LEO group (McNemar’s test; *p* < .001) and 92.6% of the EMS group answering “False” post-training (McNemar’s test; *p* < .001). This reflected an improvement of 64.6% and 55.6% among LEO and EMS participants, respectively (see Fig. 1). The proportion of correct post-training responses did not significantly differ across professional groups (Fisher’s exact test; *p = .*301).

## Discussion

With this study, we determined that a brief, 10-min presentation and discussion of accurate information regarding the lack of risk associated with incidental fentanyl exposure reduces emergency responders’ endorsement of the belief they could overdose from such contact and that such endorsements varied between LEO and EMS professional groups. Indeed, our results suggest LEOs are more likely than EMS personnel to believe they can overdose from touching fentanyl, though this difference goes away following the corrective, informational presentation. The pre-training difference is likely due to EMS personnel having more formal medical training, more experience responding to opioid overdoses than LEOs, and less interaction with police and the US Drug Enforcement Administration (DEA) colleagues who have been the driving force behind misinformation about risks of fentanyl contact [17, 31]. Overall, it is promising that both professional groups responded well to the brief informational presentation, with over 80% of LEOs and over 90% of EMS personnel demonstrating a factually accurate understanding about the lack of overdose risk from incidental fentanyl exposure in the post-training survey.

In the context of COVID-19 and the emerging threats faced by first responders, it is imperative they have accurate, up-to-date information regarding their own occupational safety. Low levels of knowledge about fentanyl, particularly among police, may reflect the relative paucity of training on occupational safety topics outside of weapons and acts of violence, as research on training pertaining to needle stick injuries has demonstrated [32].

### Limitations and future directions

Despite its novelty and important implications, our study is not without its limitations, including the lack of generalizability of these findings beyond predominantly White and male emergency responders in the Midwest, as well as the relatively small sample size and simple study design, prevented us from conducting more advanced analyses, assessing confounding variables, or drawing causal inferences. Additionally, we do not have information to link responders’ beliefs with their tangible behaviors in the field or the protocols of their agencies. For example, an individual police officer may understand incidental contact with fentanyl is innocuous but still be bound by departmental policy to don excessive protective gear or call for responder backup before attempting to save the life of the individual who has overdosed. Future research should explore associations between departmental protocols, individual responders’ concerns about fentanyl exposure, and responders’ behaviors on the scene. Also, our 2 h training (described in the “Methods” section) included a number of additional modules not directly related to fentanyl contact. As the information addressing fentanyl misconceptions was concise and required only 10 min to teach and discuss, future studies should investigate whether a brief fentanyl-focused training is sufficient to alleviate fears about fentanyl exposure.

## Conclusions

To our knowledge, this study is the first to demonstrate the effectiveness of a training intervention in correcting emergency responders’ potentially dangerous misconception that they can overdose from touching fentanyl, a misconception that could result in unnecessary delays when responding to an actual overdose, which requires urgent administration of naloxone and rescue breathing. With brain hypoxia and fatal overdose potentially occurring within minutes of one experiencing respiratory depression from potent opioid consumption [33], such delays in responding could result in the serious brain injury or death of the overdose victim. Emergency responder training programs about overdose recognition and response should include medically accurate information about the lack of danger of accidental skin contact with fentanyl to ensure responders act as quickly as possible when called to the scene of a life-threatening overdose.

## Data Availability

The datasets relevant to the current study are available from the corresponding author on reasonable request.

## Abbreviations

DEA: US Drug Enforcement Administration
EMS: Emergency medical services
LEO: Law enforcement officer
